# Impact of terminal polar substitution on elastic, electro-optic and dielectric properties of four-ring bent-core nematic liquid crystals†

**DOI:** 10.1039/c8ra00575c

**Published:** 2018-03-26

**Authors:** R. K. Khan, S. Turlapati, N. Begum, G. Mohiuddin, N. V. S. Rao, S. Ghosh

**Affiliations:** Department of Physics, University of Calcutta 92 Acharya Prafulla Chandra Road Kolkata 700 009 India sharmisthaghos@gmail.com; Chemistry Department, Assam University Silchar 788011 India

## Abstract

Here we report the influence of terminal –F, –Cl and –NO_2_ substitution on the elastic, dielectric and polar switching behavior of four-ring bent-core liquid crystals (LCs). Elastic constants of nematic liquid crystals are the key parameters in determining the threshold voltage and sensitivity to electro-optical response in a device. The elastic properties of bent-core liquid crystal systems show atypical temperature dependence and there is no hard-core theory to explain the behavior. However based on molecular simulation and atomistic calculations it is found in earlier studies that the bend angle dominates the behavior of elastic constants and the terminal or lateral substitutions have very little effect. Here we have studied three bent-core compounds which are differentiated only by their terminal polar substitution. The bend angle is identical (∼146°) for all the three compounds yet they show dramatically different elastic properties. In the fluoro-substituted compound *K*_11_ > *K*_33_, while for the other two compounds *K*_33_ > *K*_11_. Thus it is evident that the terminal polar substitution plays vital role in determining the elastic properties of bent-core systems. Correlating the mesophase ranges with the respective dipole moments of the samples it is observed that the fluoro-substituted compound (11-2M-F) with lowest dipole moment favours only nematic phase with smallest mesophase range (46.1 °C), compound 11-2M-Cl with moderate dipole moment favours short range nematic, broad range smectic with moderate mesophase range (53.1 °C), whereas the compound 11-2M-NO_2_ possesses the widest mesophase range (99.8 °C) with a very narrow nematic and a broad smectic phase amongst the three studied compounds.

## Abstract 2

Here we report the influence of terminal –F, –Cl and –NO_2_ substitution on the elastic, dielectric and polar switching behavior of four-ring bent-core liquid crystals (LCs).

## Graphical abstract

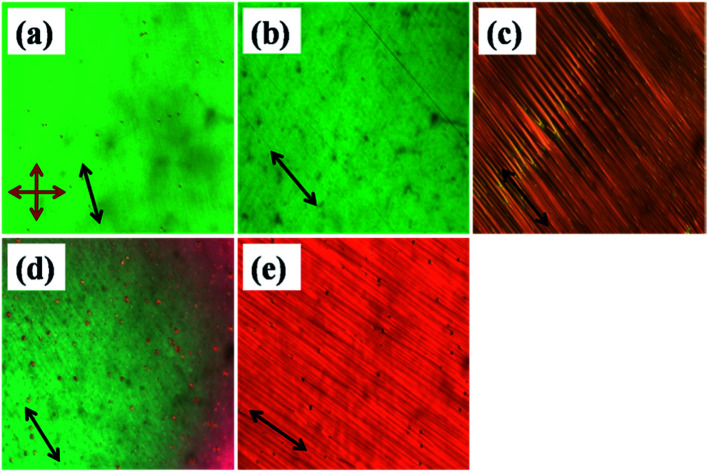

## Introduction

Liquid crystals (LCs) characterized by bent molecular motifs represent a relatively new sub-class of thermotropic mesophases. Depending upon the bend angle, chemical moieties and molecular dipoles, bent-core mesogens exhibit outstanding properties along with several new mesophases which differ significantly from mesophases of rod-like and disc-like compounds.^1–5^ Antiferroelectric and ferroelectric switching in tilted and orthogonal smectic phases composed of achiral molecules arise due to spontaneous symmetry breaking on a macroscopic scale.^6^ In recent years the nematic phase of bent-core molecules has been the topic of intensive research owing to several distinct features such as giant flexoelectric effect,^7^ ferroelectric switching,^8^ negative bent-splay elastic anisotropy^9^*etc.* It has been suggested that the observed unusual behaviour can be attributed to a high propensity of the molecules to form nano-sized smectic-C (SmC)-type clusters.^10^ A wealth of information on the structure–property relationships of different bent-core molecular architectures is described in the literature.^2,11,12^ To tune the physical properties and for achieving new mesophases various attempts have been made, such as synthesizing new compounds, preparing the mixture of bent-core/rod like^13–15^ and dispersing various type of nano/micro particles.^16–18^ Changing the position, size and length of lateral substituents on central or terminal ring can also be a new route for achieving new mesophases. Tailoring the length of the terminal chains in a five-ring bent-shaped resorcinol derivatives and alteration in the lateral substituents gives rise to new mesophases and interesting phase sequences.^19^ The nematic to isotropic clearing temperature for 1,3,4-oxadiazole-based bent-core mesogens possessing lateral methyl groups decreases with increasing the number of methyl groups and are promising candidates in the search for low temperature biaxial thermotropic nematics.^20^ A cyano substitution at the central core of achiral five-ring banana-shaped compounds results in formation of B2, SmA, and SmC phases.^19^ Electro-optical studies on the B2 phase reveal an antiferroelectric/ferroelectric switching.^21^ The influence of lateral substituents on the central core and (or) on the terminal rings is much more pronounced for bent-core compounds than for the comparable calamitic cases. For resorcinol derivatives, Cl, Br and CH_3_ substituents in the terminal-position of the outer rings and F, Cl, CN, NO_2_, CH_3_ at different positions of the central ring depress the clearing temperature and vary the bend angle.^22^ Occurrence and the thermal stabilization of the nematic phase mainly depend upon the nature and position of substitutions on the central core and the two rigid arms, as well as the type of linkage units. Again the nature and structure of the liquid crystalline phases of photosensitive bent-core mesogens containing five aromatic rings with 4-chlororesorcinol, 4-bromoresorcinol or 4-fluororesorcinol as the central bent unit, are greatly affected by the nature and the polarity of the substituents on the central benzene ring.^23^

Elastic constants of nematic liquid crystals are the key parameters in determining the threshold voltage and sensitivity to electro-optical response in a device. The elastic properties of bent-core liquid crystal systems show atypical temperature dependence and there is no hard-core theory to explain the behavior. However based on molecular simulation and atomistic calculations it is found in earlier studies that the bend angle dominates the behavior of elastic constants and the terminal or lateral substitutions have very little effect.^24^

In this paper we report the effect of terminal polar substitution (–F, –Cl, –NO_2_) on the elastic, polar switching and dielectric behavior of a four-ring bent-core liquid crystals with identical bend angle. The elastic properties change significantly for different terminal substituents. The splay (*K*_11_) elastic constant is greater than the bend (*K*_33_) elastic constant for the fluorine-substituted compound throughout the nematic phase similar to other bent-core compounds observed earlier.^25–28^ However *K*_33_ ≫ *K*_11_ for the other two compounds with chlorine and nitro substitutions, comparable to calamitic LCs. We conclude that the terminal substitution plays important role in determining the elastic constants. Dielectric and electro-optical (E-O) studies reveal the existence of cybotactic clusters in the nematic phases of the compounds.

## Results and discussion

### Optical investigation

**Fig. 1:**
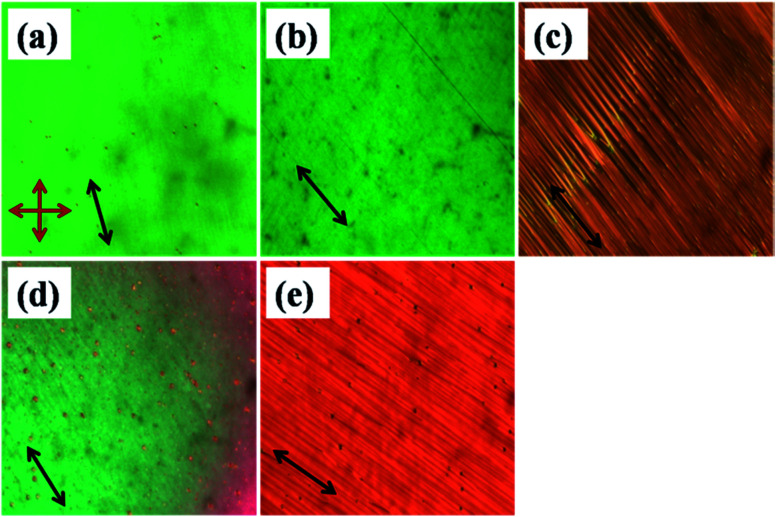
Optical micrographs in 5 μm planar cell of (a) 11-2M-F in nematic phase at 135 °C, (b) 11-2M-Cl in nematic phase at 145 °C, (c) striped SmA phase of 11-2M-Cl at 135 °C, (d) nematic phase of 11-2M-NO_2_ at 170 °C and (e) striped SmA phase of 11-2M-NO_2_ at 112 °C (the black arrow shows the rubbing direction and red arrows represent the orientations of the analyzer and polarizer).

The optical micrographs of various mesophases of the compounds are shown in Fig. 1(a–e). 11-2M-F exhibits an enantiotropic nematic (N) phase (Fig. 1(a)) whereas the other two compounds 11-2M-Cl (Fig. 1(b) and (c)) and 11-2M-NO_2_ (Fig. 1(d and e)) possess additional smectic phases beneath the nematic phase. The fluoro-substituted compound has the highest nematic range. However the clearing temperature and mesomorphic range increases in the chloro-substituted compound (11-2M-Cl) and becomes highest in the compound with the strong electron-withdrawing nitro group (11-2M-NO_2_). Quantum chemical calculations based on density functional theory (DFT) reveal that the dipole moment is lowest for the compound 11-2M-F, moderate for 11-2M-Cl and highest for 11-2M-NO_2_ (ESI, Table S1†). Thus correlating the mesophase ranges with the respective dipole moments of the samples it is evident that the compound with lowest dipole moment favours only nematic phase with smallest mesophase range (46.1 °C), compound 11-2M-Cl with moderate dipole moment favours short range nematic, broad range smectic with moderate mesophase range (53.1 °C), whereas the compound 11-2M-NO_2_ possesses the widest mesophase range (99.8 °C) with very narrow nematic and a broad smectic phase amongst the three studied compounds (Table 1).

**Table 1:** Phase transition temperatures (°C) and liquid crystalline phase thermal range of the compounds 11-2M-F, 11-2M-Cl and 11-2M-NO_2_ recorded for second heating (first row) and second cooling (second row) cycles at 5 °C min^−1^ from DSC and confirmed by polarized optical microscopy. The enthalpies (Δ*H* in kJ mol^−1^) and entropies (Δ*S* in J mol^−1^ K^−1^) respectively are presented in parentheses. *n* represent the number of methylene units in the end alkyloxy chains

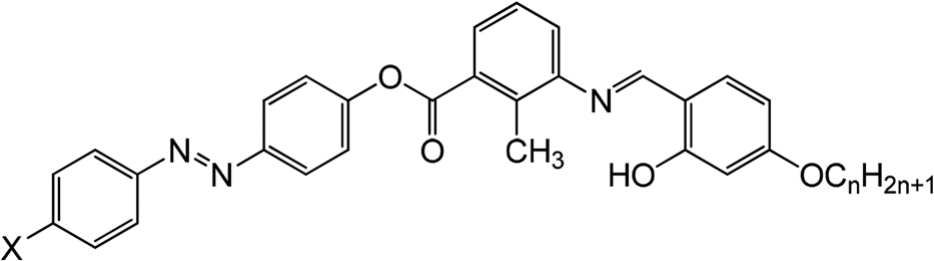		
Compound	X	Phase transition temperatures in °C (enthalpy, entropy)
11-2M-F	F	Cr 119.4 (44.8, 114.2) 136.9 (0.348, 0.85) Iso
		Cr 90.4 (41.1, 113.2) N 136.5 (0.328, 0.80) Iso
11-2M-Cl	Cl	Cr 138.9 (44.4, 107.9) N 154.9 (0.334, 0.78) Iso
		Cr 101.0 (36.5, 97.6) SmA 136.9 (0.246, 0.60) N 154.1 (0.226, 0.52) Iso
11-2M-NO_2_	NO_2_	Cr 92.9 (22.5, 61.6) SmA 171.6 (0.40, 0.09) N 175.8 (0.33, 0.74) Iso
		Cr 73.6 (20.9, 60.4) SmA 169.1 (0.43, 0.98) N 173.4 (0.31, 0.70) Iso

Fig. 1(c) and (e) exhibit striped pattern indicating conventional uniaxial smectic A phase with optic axis lying along the rubbing direction. In 11-2M-NO_2_ nematic droplets appear at isotropic to nematic phase transition which coalesces to form uniform texture in planar cell (Fig. 1(d)) as the sample is cooled. The delicate balance between the size and polarity of the substituents determines the packing of the molecules and hence the mesomorphic range.

### Dielectric anisotropy and elastic constants

**Fig. 2:**
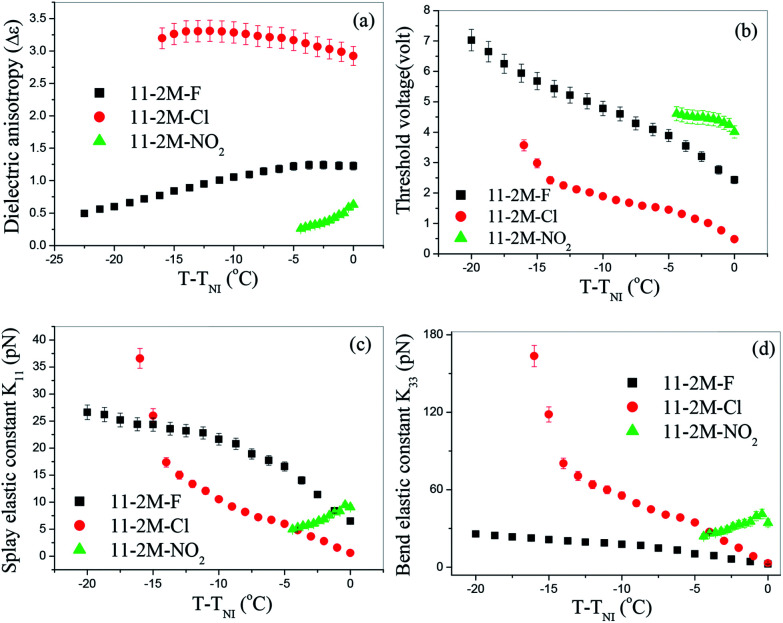
Variation of (a) dielectric anisotropy (Δ*ε* = *ε*_‖_ − *ε*_⊥_), (b) threshold voltage (*V*_th_), (c) splay elastic constant (*K*_11_) and (d) bend (*K*_33_) elastic constant as a function of reduced temperature (*T* − *T*_NI_). The error bar corresponds to 5% experimental error.

The threshold voltage, parallel and perpendicular components of dielectric permittivity are obtained from analysis of the variation in capacitance of a planar device that results from the electric-field induced Freedericksz transition at 5 kHz. Fig. 2(a) exhibits the variation of dielectric anisotropy (Δ*ε*) as a function of reduced temperature (*T* − *T*_NI_).

All compounds possess positive dielectric anisotropy throughout the nematic phase while chloro-substituted compound has the highest magnitude. This large value of Δ*ε* suggests that substitution of Cl– atom in place of F– atom or NO_2_-group enhances the lateral dipole of the molecule and hence increases the dielectric anisotropy. However this fact is not consistent with the lateral dipole moments of the molecules obtained from DFT calculations where *μ*_X_ is highest for 11-2M-NO_2_ (∼10 D), intermediate for 11-2M-Cl (∼5.91 D) and lowest for 11-2M-F (∼5.12 D). Here we conjecture that owing to the high dipole moment of 11-2M-NO_2_, the molecules tend to organize in an anti-parallel fashion to form a dimer and thus minimize the free volume and resultant dipole moment of the bulk system. We have calculated the binding energies of the most probable dimer conformations of 11-2M-F, 11-2M-Cl and 11-2M-NO_2_*via* structure optimization using DFT calculation, and found that the binding energy is highest for 11-2M-NO_2_ (ESI, Table S3†). Hence among the three compounds 11-2M-NO_2_ endorses the most strong dipole–dipole interaction in an antiparallel molecular arrangement to provide the stability of dimeric arrangement with respect to temperature in the respective mesophases. This antiparallel arrangement of 11-2M-NO_2_ molecules reduces the parallel component of permittivity (*ε*_‖_) significantly and consequently Δ*ε* becomes small. On the other hand the binding energy for formation of dimers in the chlorinated and fluorinated compound is relatively small and has fewer tendencies to form dimers. Hence 11-2M-Cl possesses the highest value of lateral dipole moment in the nematic mesophase.

The magnitude of Δ*ε* of 11-2M-Cl enhances with decreasing temperature similar to typical calamitic NLCs, but tends to decrease slightly near nematic-smectic transition temperature (*T*_SN_).^15,36^ For 11-2M-F, Δ*ε* decreases as the sample is cooled from isotropic phase. This non-monotonous behavior of Δ*ε* can be explained by the decreasing nature of *ε*_‖_ owing to increasing size of cybotactic clusters. Among these smectic-like clusters the inter-molecular separation within the smectic layers is much smaller than that in the direction perpendicular to the layers. Hence the molecules within a smectic layers have tendency of arranging in antiparallel manner. This reduces the parallel component of dielectric permittivity while the perpendicular component increases as all the dipole moments along the bow axis line up in the same direction.^37^ Similar arguments can explain the small decreasing behavior of Δ*ε* on cooling for narrow nematic range of 11-2M-NO_2_. Jang *et al.* observed significant changes in dielectric anisotropy in three bent-core nematic liquid crystals having the same core but with different chain length at terminal position. The molecule with short tail exhibited sign reversal of Δ*ε* whereas the longest molecule revealed negative dielectric anisotropy in the entire nematic phase. Thus modifications at terminal position of bent-core molecules influences the dielectric anisotropy of the medium significantly.

Threshold voltage is greatly influenced by the dielectric anisotropy of the system which determines the interaction strength of the molecules with applied electric field. The dependence of *V*_th_ on reduced temperature for all compounds is shown in Fig. 2(b). At a common temperature *T* − *T*_NI_ = −4 °C, *V*_th_ is highest for 11-2M-NO_2_ ∼4.5 V, while it is lowest for 11-2M-Cl ∼1.3 V (Table 2).

**Table 2:** Dielectric anisotropy, threshold voltage and elastic parameters for compounds 11-2M-F, 11-2M-Cl and 11-2M-NO_2_

Compounds	Δ*ε*	*V*_th_ (V μm^−1^)	*K*_11_ (pN)	*K*_33_ (pN)
11-2M-F	1.2	3.6	14.4	9.3
11-2M-Cl	3.1	1.3	4.8	27.3
11-2M-NO_2_	0.3	4.5	5.3	26.1

To understand the elastic properties of the compounds splay (*K*_11_) and bend (*K*_33_) elastic constants are plotted separately in Fig. 2(c) and (d). *K*_11_ for 11-2M-F varies from ∼6.5 pN near *T*_NI_ up to a maximum value ∼26.6 pN. For the nitro compound *K*_11_ decreases continuously on cooling and possess relatively small magnitude (9–4.9 pN). Lee *et al.* reported similar decrease in splay elastic constant at low temperature side in mixtures of rod-like/bent-core LCs and the effect was pronounced when higher percentage of bent-core molecules are added.^42^ On the contrary, the compound 11-2M-Cl exhibits typical linear increasing behaviour of *K*_11_ upon cooling as observed in a number of oxadiazole based bent-core materials.^24,27^ It takes the value 0.6 pN close to *T*_NI_ and increases continuously up to 17.5 pN at *T* − *T*_NI_ = −14 °C and then diverges near nematic-smectic transition. Fig. 2(d) exhibits the behavior of *K*_33_ which is generally expected to increase on cooling.^9,24,28^ The bend elastic constant is lowest for 11-2M-F and increases (2.4–24.7 pN) up on cooling. The highest magnitude (3.1–163.5 pN) of *K*_33_ is observed for 11-2M-Cl which increases sharply on reducing temperature followed by a divergence at the end near *T*_SN_. 11-2M-NO_2_ displays non-monotonous variation of *K*_33_ over temperature. *K*_33_ first increases and then decreases as the sample is cooled from its isotropic temperature. For such reduction of *K*_33_ at the low temperature region of nematic phase is important with respect to formation of twist-bend nematic phases.^38^ To present a clear view of the elastic behavior the values of Δ*ε*, *V*_th_, *K*_11_ and *K*_33_ for all the samples at *T* − *T*_NI_ = −4 °C is shown in Table 2 for comparison.

The ratio of splay to bend elastic constant (*K*_11_/*K*_33_) is <1 for the compounds 11-2M-Cl and 11-2M-NO_2_. For 11-2M-Cl, *K*_11_/*K*_33_ varies from ∼0.19 near *T*_NI_ to ∼0.17 at *T* − *T*_NI_ = −5 °C and again rises to ∼0.22 near nematic to smectic transition. In 11-2M-NO_2_, *K*_11_/*K*_33_ varies between ∼0.2–0.26 over the nematic range. On the contrary *K*_11_/*K*_33_ is >1 at all temperatures for 11-2M-F and varies from ∼2.63 near *T*_NI_ to ∼1.03 at near nematic to crystal transition. In low molecular mass rigid-rod-like liquid crystals, it is both theoretically predicted and experimentally established that bend elastic constant is much higher than splay elastic constant, *i.e.*, *K*_11_/*K*_33_ is <1.^39^ However most of the BCNs reported so far exhibit *K*_11_/*K*_33_ is >1. Kundu *et al.* observed for the first time in a mixture of rod-like and bent-core molecules that *K*_11_/*K*_33_ < 1 for pure rod-like LC but the scenario gets opposite (*K*_11_/*K*_33_ is >1) after bent-core molecules are added.^15^ Sathyanarayana *et al.* found *K*_11_/*K*_33_ ∼ 1.42 near *T*_NI_ then increases to 2.5 at *T* − *T*_NI_ = −45 °C followed by a decrease owing to pretransitional divergence of *K*_33_.^25^ Tadapatri *et al.* observed *K*_11_/*K*_33_ > 1 at all temperatures for a cyanoresorcinol-based five-ring bent-core LC.^28^ Majumdar *et al.* reported *K*_11_/*K*_33_ ∼ 3.5 at *T* − *T*_NI_ = −12 °C and attributed the softening of *K*_11_ as compared to *K*_33_ to formation of cybotactic clusters.^26^ Kaur *et al.* obtained *K*_11_/*K*_33_ between 2.5 near TNI to 1.25 at *T* − *T*_NI_ = −55 °C.^27^ In contrast, only few bent-core nematics are there with *K*_11_/*K*_33_ < 1 similar to calamitic liquid crystals.^24,40^

In summary *K*_11_ > *K*_33_ for 11-2M-F at all temperatures as observed for most BCNs, but for the other two compounds *K*_33_ > *K*_11_ similar to rod-like LCs. In a thiadiazole based bent-core nematic, bend elastic constant was found to be greater than splay elastic constant and the effect was attributed to extended bend angle (164°).^24(b)^ On the other hand, the bent-core oxadiazole compounds which exhibit typical elastic behavior (*K*_11_ > *K*_33_) of bent-core systems possess bend angle ∼140°.^41^ The studies suggested that, not the lateral or terminal chains but the bend angle dominates the elastic behavior of the bent-core systems.^24^ Salamon *et al.* reported another thiadiazole based BCN with *K*_33_ > *K*_11_ and the non-BCN behavior was assigned to the four alkyl chains connected to intermediate core rings which act like soft spacers between molecules and prevents close polar packing.^41^ However in our case the bend angle is identical (∼146°) for all the compounds^29,30^ and hence it cannot be the dominant parameter in governing the elastic constants. Also the molecules do not have any lateral alkyl chains which may prohibit formation of polar clusters. In fact 11-2M-Cl shows polar response in nematic phase which is related to the switching of the cybotactic clusters. Quantum chemical calculations suggest that the polarizability and dipole moment components change significantly after terminal polar substitution (ESI, Tables S1 and S3†) and this in turn is expected to alter the inter- and intramolecular interactions. Since elasticity originates from molecular interactions, it is evident that the terminal substitution plays important role in determining the elastic properties of the compounds under study. Another possibility that can be considered is the underlying smectic A phase. Both compounds 11-2M-Cl and 11-2M-NO_2_, which show *K*_33_ > *K*_11_, have smectic A phase that could have influenced the elastic properties of the compounds.^41^ The chlorinated compound shows strong pretransitional divergence for *K*_11_ and *K*_33_ similar to earlier report on a hockey-stick shaped molecule with low lying SmA phase.^13^

### Spontaneous polarization

**Fig. 3:**
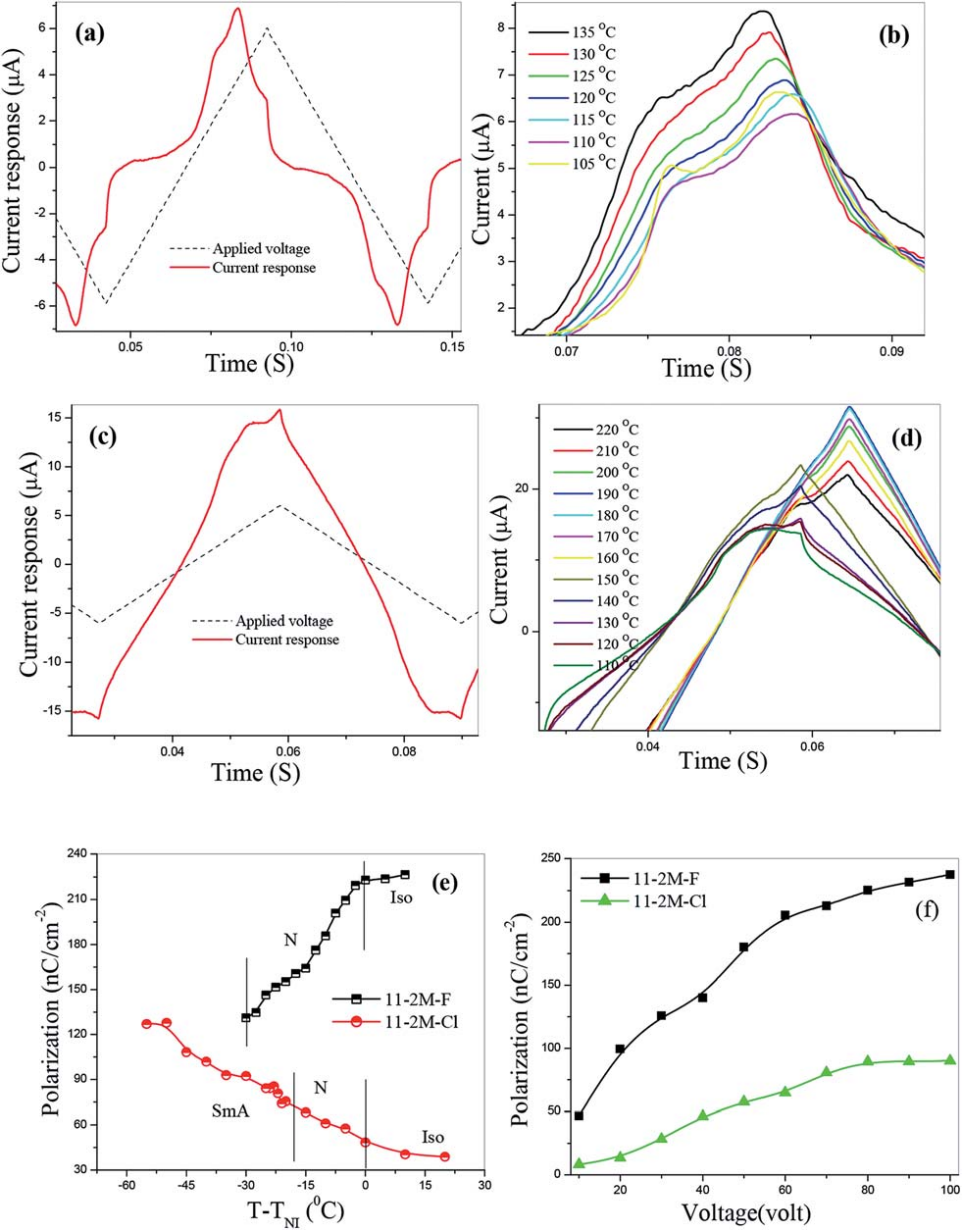
Investigation of current response applying triangular wave voltage 20 V_PP_ μm^−1^, 10 Hz for (a) 11-2M-F at 120 °C; (b) 11-2M-F at different temperatures; (c) 11-2M-Cl at 130 °C; (d) 11-2M-Cl at different temperatures; (e) temperature dependence of spontaneous polarization for 11-2M-F and 11-2M-Cl and (f) voltage variation of spontaneous polarization for 11-2M-F and 11-2M-Cl.

Spontaneous polarization is measured in two compounds 11-2M-F and 11-2M-Cl in entire nematic and smectic phase. A prominent current peak per half cycle of the applied triangular voltage was observed for 11-2M-F in the entire nematic phase (Fig. 3(a) and (b)). The broad peak indicates the overlapping of two peaks of different origin and hence implies antiferroelectric ordering. The peak at left which becomes prominent at lower temperature must have polar origin whereas the other one may have both polar and ionic origin. The other compound 11-2M-Cl does not possess any prominent peak instead a small bump on the applied voltage arm is observed (Fig. 3(c) and (d)). The area under the current response peak is proportional to spontaneous polarization (*P*_S_) and its temperature dependence is shown in Fig. 3(e). The observation of spontaneous polarization in nematic phase of 11-2M-F is attributed to the switching of the cybotactic clusters. As the sample is cooled from isotropic, spontaneous polarization (*P*_S_) decreases from the highest saturated value 222 nC cm^−2^ to the lowest value 131 nC cm^−2^ near N–Cr transition. The observed behavior can be attributed to participation of less number of clusters in switching mechanism at lower temperature due to strong increase in viscosity. For the material 11-2M-Cl, in the isotropic phase *P*_S_ is almost constant and has very low magnitude (∼38 nC cm^−2^). As the sample is cooled *P*_S_ increases through the nematic phase and also in the underlying SmA phase and takes the maximum value 127 nC cm^−2^. The voltage variation shows typical enhancement of *P*_S_ upon increasing magnitude of applied voltage (Fig. 3(f)) and subsequent saturation of the value confirming the polar origin of the current peaks. The spontaneous polarization shows opposite temperature dependence for the fluoro- and chloro-substituents. *P*_S_ of the compound 11-2M-F decreases on cooling. Similar atypical trend has been observed in its lower homologue 6-2M-F^31^ and 7-2M-F^32^ with no underlying smectic phase. However in its higher homologue 16-2M-F with low temperature smectic A phase, *P*_S_ increases on cooling in the nematic and SmA phase. Similar conflicting behaviour of *P*_S_ has also been observed in nematic phase of lower and higher homologues of the chlorinated compounds 6-2M-Cl (Cr 88.5 N 165.5 Iso)^31^ and 11-2M-Cl with low lying SmA phase. Hence it is evident that underlying smectic phase influences the nature of temperature dependence of *P*_S_ of the compounds. The nitro compound does not show any polar switching peak in any mesophases (ESI, Fig. S2†).

### Dielectric spectroscopy

**Fig. 4:**
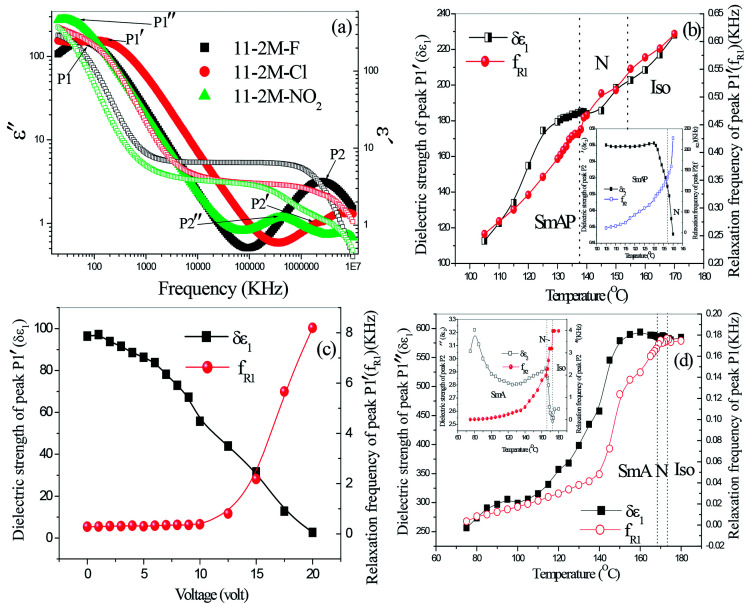
(a) Dielectric spectra at 130 °C of the compounds 11-2M-F, 11-2M-Cl and 11-2M-NO_2_; (b) temperature dependence of dielectric strength (*δε*) and relaxation frequency (*f*_R_) of peak P1′ and P2′ (inset) of 11-2M-Cl, (c) effect of bias voltage on *δε* and *f*_R_ of P1′, (d) temperature dependence of dielectric strength (*δε*) and relaxation frequency (*f*_R_) of peak P1′′ and P2′′ (inset) of 11-2M-NO_2_.

Dielectric spectroscopy revealed information about the molecular relaxation processes in the compounds. A typical dielectric spectra of 11-2M-F shows two peaks in the entire nematic range (Fig. 4(a)) as observed earlier for its lower homologues (*n* = 6, 7).^31,32^ The high frequency (2 MHz) peak P2 corresponds to a molecular mode occurring due to rotation of the molecules along their long axes whereas other peak P1 appears at extremely low frequency (50 Hz) range where the polarization peak appears in E-O studies. P1 is collective mode and appears as a result of cooperative movement of the molecules in cybotactic clusters. This kind of peak has been observed earlier in other bent-core nematic possessing cybotactic clusters.^33^ In 11-2M-Cl similar relaxation peaks are present in the nematic and smectic phases (Fig. 4(a)), however the peak at left is at relatively higher frequency (∼250 Hz) indicating lower viscosity of the material. The dielectric strengths (*δε*) and relaxation frequencies (*f*_R_) of the peaks are obtained by fitting the dielectric spectra with Havriliak–Negami fitting function^34^ and their temperature dependence are shown in Fig. 4(b). Both *δε* and *f*_R_ of peak P1′ decreases upon cooling owing to increase in viscosity and subsequent difficulties in movement of cybotactic clusters. For the high frequency peak P2′, *δε* increases abruptly as the sample is cooled from isotropic to smectic phase *via* nematic phase and finally gets saturated whereas *f*_R_ decreases continuously on cooling. To ascertain the collective nature of the low frequency mode we employed bias voltage. The dielectric strength decreases with increasing bias voltage and suppressed at 20 V (Fig. 4(c)). Relaxation frequency remains constant up to 10 V and then increases rapidly. The dielectric behavior of 11-2M-NO_2_ is shown in Fig. 4(d).

## Experimental

The bent-core compounds 11-2M-F, 11-2M-Cl and 11-2M-NO_2_ are synthesized following a very simple and straightforward methodology of the standard procedures described elsewhere.^29,30^ The unsymmetrical four-ring molecules possess an alkoxy chain attached at only one end of the bent-core molecule, while the other arm ends with a highly polar fluoro/chloro/nitro substituent. The end polar substituent in one of the arms contributes a large dipole moment. The thermal behavior of the compounds *i.e.* transition temperatures, and associated transition enthalpies were investigated using a differential scanning calorimeter and are shown in Table 1. For further confirmation of the phase transition temperature we used polarized optical microscopy using a Leica DMLP microscope.

All dielectric and electro-optic measurements were carried out using planar cells of thickness 5 μm (Instec Inc. USA) and 4 μm (EHC Japan) with antiparallel alignment. The temperature of the cells are controlled precisely (±0.1 °C) using Instec HCS-302 hot stage. The cells are filled by capillary action at temperature well above the nematic–isotropic transition temperature of the compounds.

The dielectric anisotropy was measured as a function of temperature in the entire nematic range using an impedance gain analyzer E4990A (Keysight technologies) at frequency 5 kHz. Electric field-induced Freedericksz transition was employed to measure the voltage dependence of capacitance (*C*–*V*) applying bias voltage with ac superposition (1 V, 5 kHz) for eliminating the ionic contribution in 4 μm cells (EHC, Japan). The applied voltage was sufficient to reorient the molecules. The bias voltage was ramped from 0 to 30 V in 0.1 V step. At low voltages below threshold, LC molecules were aligned along the rubbing direction *i.e.* perpendicular to the applied electric field direction. The resultant capacitance and dielectric permittivity measured at this planar geometry are denoted as *C*_⊥_ and *ε*_⊥_. The parallel components (*C*_‖_ and *ε*_‖_) were measured by extrapolating the voltage dependency of capacitance in the high voltage range (20–30 V) in homeotropic condition where the molecules are oriented parallel to the electric field direction. The dielectric anisotropy is calculated as Δ*ε* = *ε*_‖_ − *ε*_⊥_.

The splay (*K*_11_) elastic constant is obtained by measuring the Freedericksz threshold (*V*_th_), using the formula1
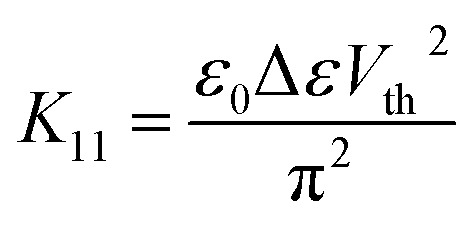


Freedericksz threshold was determined optically by monitoring the transmitted light intensity of a He–Ne laser (*λ* = 632.8 nm) from the planar cell of thickness 4 μm as a function of applied voltage.

Following Uchida's approach,^35^ bend (*K*_33_) elastic constants is extracted by fitting the experimental data well above *V*_th_ with the expression:2
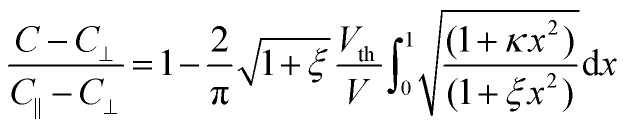
where 
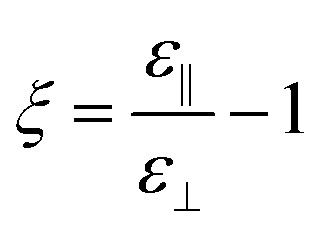
 and 
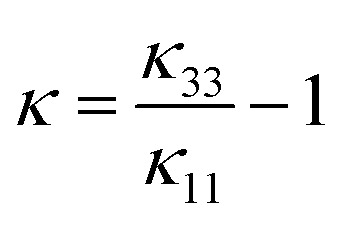
.

Spontaneous polarization was measured using triangular wave method in a 5 μm planar cell. HP 33120A signal generator (Hewlett Packard), an F10A voltage amplifier (FLC Electronics, Sweden) and a DL1620 oscilloscope (Yokogawa, Nakacho, Musahino, Tokyo, Japan) were employed for the measurement. Dielectric spectroscopy was done using the impedance gain analyzer E4990A (Keysight technologies), in the frequency range 20 Hz to 10 MHz.

## Conclusions

In this paper we describe the influence of terminal –F, –Cl and –NO_2_ substitution on the elastic, dielectric and polar switching properties of four-ring bent-core liquid crystals. Dielectric anisotropy is highest for the compound 11-2M-Cl and exhibits normal temperature dependence whereas the other two compounds show unusual behavior on cooling. Dielectric anisotropy is positive for all the compounds and the non-monotonous temperature dependence can be attributed to the formation of cybotactic clusters. For the compound 11-2M-F, *K*_11_ > *K*_33_ as observed in most BCNs, while *K*_33_ > *K*_11_ for the other two compounds. Our conclusion is, not the bend angle but the conformational change of the molecules resulting from terminal substitution is the dominant factor in determining the elastic constants. Also the compounds which exhibit calamitic like behaviour of the elastic constants (*K*_33_ > *K*_11_) both have underlying SmA phase. Hence the low lying smectic phase may have influence on the elastic properties of the compounds. Pretransitional divergence is observed for both splay and bend elastic constants in one of the compounds, 11-2M-Cl, near nematic-smectic A transition. Dielectric spectroscopy reveals a low frequency peak in the nematic phase for all compounds corresponding to short-scale cybotactic clusters. The collective nature of this peak is determined applying bias voltage. E-O studies reveal prominent current peak per half cycle of the applied triangular voltage for the fluorine and chlorine substituted compounds. Spontaneous polarization increases on cooling and becomes highest in the smectic A phase for 11-2M-Cl whereas it show opposite behavior for 11-2M-F. This conflicting temperature dependence of *P*_S_ is linked with the underlying smectic phase. In summary the terminal polar substitution plays important role in determining the elastic, dielectric and E-O properties and packing of four-ring bent-core molecules with identical bend angles.

## Conflicts of interest

There are no conflicts to declare.

## Supplementary Material

RA-008-C8RA00575C-s001
