# Supplementary material for: Impact of terminal polar substitution on elastic, electro-optic and dielectric properties of four-ring bent-core nematic liquid crystals
Source: RSC Adv. 2018 Mar 26;8(21):11509–16. doi: 10.1039/c8ra00575c (PMC9079133; doi:10.1039/c8ra00575c)
Supplement: RA-008-C8RA00575C-s001 [file RA-008-C8RA00575C-s001.pdf]

Electronic Supplementary Material (ESI) for RSC Advances.
This journal is © The Royal Society of Chemistry 2018

Supplementary Material for Publication

Impact of terminal polar substitution on elastic, electro-optic and dielectric properties of four-ring bent-core nematic liquid crystal

R. K.Khan[1], S.Turlapati[2], Nazma Begum[2], Golam Mohiuddin[2], N. V. S. Rao[2], and S. Ghosh[1*]

[1]Department of Physics, University of Calcutta, 92 Acharya Prafulla Chandra Road, Kolkata 700 009, India, email: sharmisthaghos@gmail.com

[2]Chemistry Department, Assam University, Silchar 788011, India.

1.1 Density functional theory calculations: The quantum mechanical calculations of molecular properties were performed using density functional theory (DFT) [1], by employing the combination of Becke3–Lee–Yang–Parr (B3LYP) hybrid functional and 6-311G(d,p) basis set using the Gaussian 09 package, to obtain the information related to molecular conformation, bending angle, dipole moment, molecular polarisability and asymmetry parameter of all the compounds. Full geometry optimizations have been carried out without imposing any constraints [1]. Spin-restricted DFT calculations were carried out in the framework of the generalized gradient approximation (GGA) by using B3LYP hybrid functional, exchange-correlation functional and the 6-311G (d, p) standard basis set [2,3]. The detailed study is reported in earlier reports [4,5]. The values of the dipole moment components, the polarizability components, asymmetry parameter etc. are summarized in table S1 and S2.

**Table S1. DFT-calculated bending angle in degrees, dipole moment components (μ_X, μ_Y, μ_Z) and the resultant dipole moment (μ) in Debye.**					
**Compound**	μ_X	μ_Y	μ_Z	$\mu_{resultant} = (\mu_X^2+\mu_Y^2+\mu_Z^2)^{1/2}$	Bending angle (°) θ
**11-2M-F**	**5.12**	**3.32**	**2.49**	**6.60**	**146**
**11-2M-Cl**	**5.91**	**3.49**	**2.51**	**7.31**	**146**
**11-2M-NO_2**	**10.00**	**4.66**	**2.53**	**11.32**	**146**

**Table S2. DFT calculated principal polarizability components (α_{XX}, α_{YY}, α_{ZZ}), isotropic component $\alpha^{iso} = (\alpha_{XX} + \alpha_{YY} + \alpha_{ZZ})/3$, anisotropy $\Delta\alpha = [\alpha_{XX} - (\alpha_{YY} + \alpha_{ZZ})/2]$, and asymmetry, $\eta = [(\alpha_{YY} - \alpha_{ZZ})/ (\alpha_{XX} - \alpha^{iso})]$.**						
**Compound**	α_{XX}	α_{YY}	α_{ZZ}	α iso	$\Delta\alpha$	η_α
**11-2M-F**	**1008**	**380**	**319**	**568**	**656**	**0.13**
**11-2M-Cl**	**1060**	**388**	**321**	**590**	**706**	**0.14**
**11-2M-NO_2**	**1082**	**397**	**323**	**600**	**722**	**0.15**

Calculation of binding energy for formation of dimers:

Polar liquid crystalline molecules having terminal polar moiety *viz.*, CN, NCS, NO_2, F, Cl, OCF_3 etc. tends to organize in an anti-parallel fashion to minimize the free volume and resultant dipole moment of the bulk system.[6,7] Hence the most probable dimer of 11-F, 11-Cl and 11-NO_2 studied *via* structure optimization using DFT calculation to obtain various information related to molecular conformations. Optimized geometry of the most probable dimers of 11-F, 11-Cl and 11-NO_2 exhibited several unconventional intermolecular hydrogen bonds (O···H–C type) present in the molecules as shown in Figure 1. The binding energy and

the resultant dipole moment calculated for all the dimers are presented in Table 1. Upon increasing the polarity of the terminal moiety (from F to Cl to NO_2) the binding energy is increasing consistently indicating the strong interactions in antiparallel conformation *via* weak H-bonding interaction between the two monomers. For compound 11-F, 11-Cl and 11-NO_2 the dipole moment is decreased by 5.1, 5.3 and 6.7 Debye unit respectively from monomer unit to dimer unit indicates that the antiparallel arrangement is becoming stronger upon increasing the polarity of the terminal substituent. Hence among the three compounds 11-NO_2 endorses the most strong dipole–dipole interaction in an antiparallel molecular arrangement to provide the stability of dimeric arrangement in the respective mesophase.

Table S3. All resultant dipole moment (Debye) in monomer and dimer and binding energy in dimer form.

**Compound**	**$\mu_{resultant}$ (monomer)**	**$\mu_{resultant}$ (dimer)**	**Binding energy (kJ/mol)**
**11-F**	**6.6**	**1.5**	**23.46**
**11-Cl**	**7.3**	**2.0**	**25.48**
**11-NO2**	**11.3**	**4.6**	**28.57**

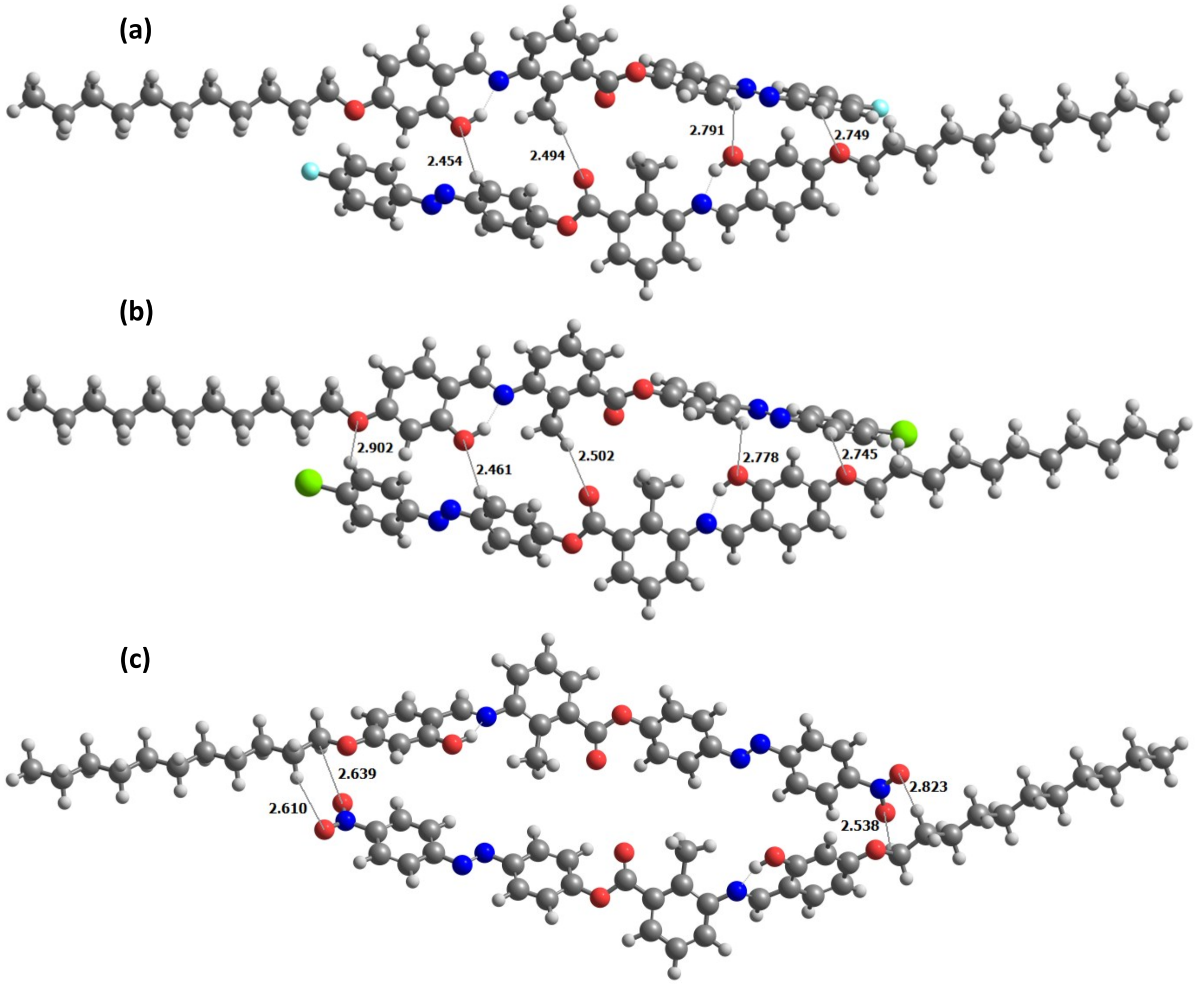

Figure S1. Antiparallel arrangement of (a) 11-F, (b) 11-Cl and (c) 11-NO_2. All the numerical presented the H-bonding are in Å unit.

1.2 Current response of 11-2M-NO_2:

We could not observe any polar peak after applying triangular wave voltage up to $100V_{PP}$ 10Hz.

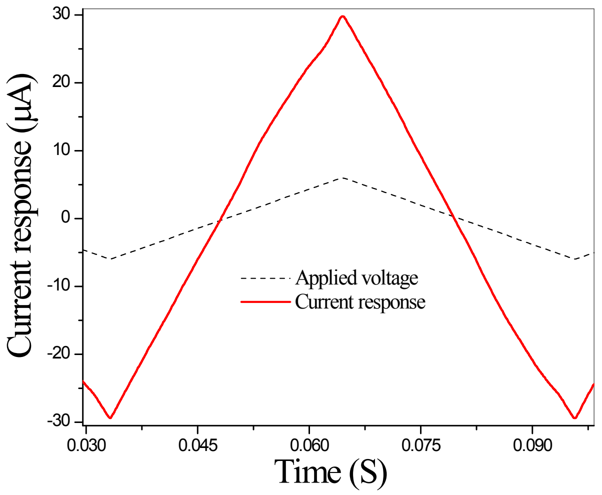

Figure S2. Investigation of current response applying triangular wave voltage $20V_{PP}$ μm^{-1}, 10 Hz for 11-2M-NO2 at 171 ^{O}C.

1.3 Dielectric spectroscopy of 11-2M-NO_2: Dielectric spectroscopy reveals two peaks in all the mesophases Peak P1 arises due to collective motion of the molecules in the nematic and smectic phases while P2 is molecular mode owing to rotation of the molecules along the long axis.

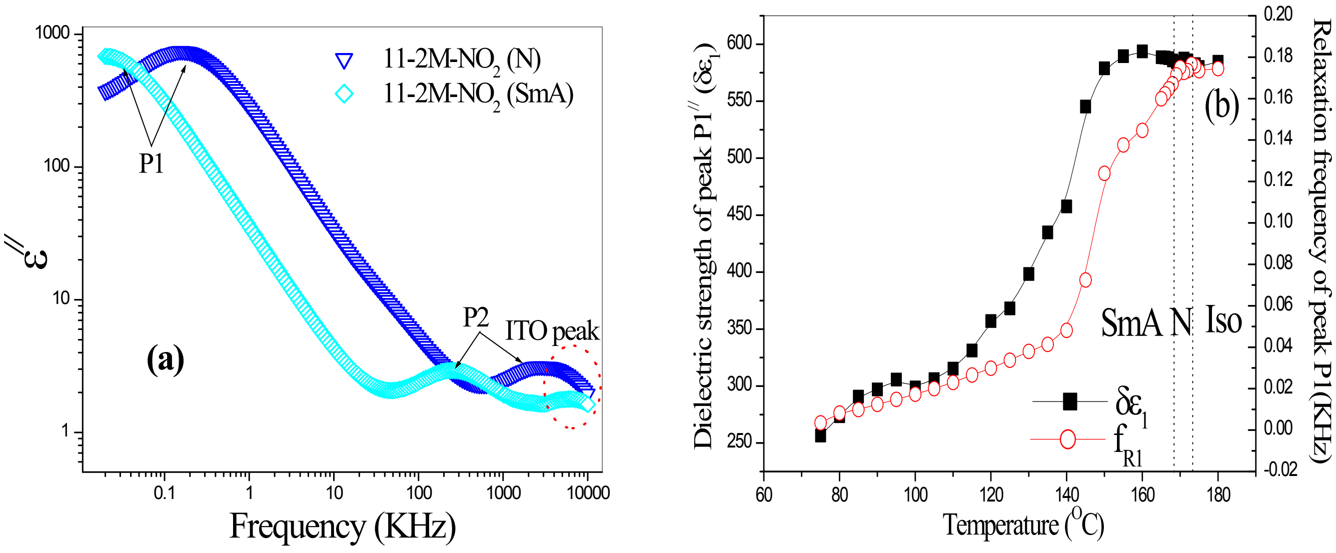

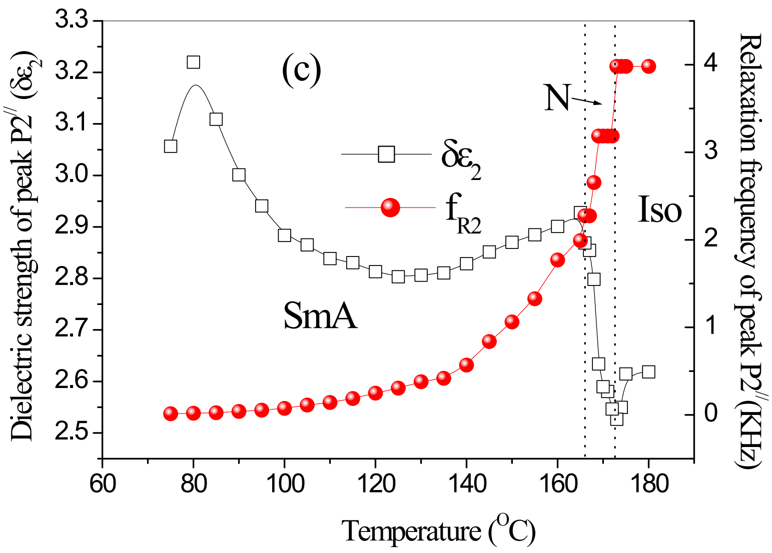

Figure S3. Investigation of the compounds **11-2M-NO$_2$** (a) dielectric spectra at 171 ^{O}C and 125 ^{O}C; temperature dependence of dielectric strength (δε) and relaxation frequency (f_R) of (b) peak P1 and (c) peak P2.

References:

1. Frisch MJ, Trucks GW, Schlegel HB, Scuseria GE, Robb MA, Cheeseman JR, Scalmani G, Barone V, Mennucci B, Petersson GA, Nakatsuji H, Caricato M, Li X, Hratchian HP, Izmaylov AF, Bloino J, Zheng G, Sonnenberg JL, Hada M, Ehara M, Toyota K, Fukuda R, Hasegawa J, Ishida M, Nakajima T, Honda Y, Kitao O, Nakai H, Vreven T, Montgomery JA Jr, Peralta JE, Ogliaro F, Bearpark M, Heyd JJ, Brothers E, Kudin KN, Staroverov VN, Kobayashi R, Normand J, Raghavachari K, Rendell A, Burant JC, Iyengar SS, Tomasi J, Cossi M, Rega N, Millam NJ, Klene M, Knox JE, Cross JB, Bakken V, Adamo C, Jaramillo J, Gomperts R, Stratmann RE, Yazyev O, Austin AJ, Cammi R, Pomelli C, Ochterski JW, Martin RL, Morokuma K, Zakrzewski VG, Voth GA, Salvador P, Dannenberg JJ,Dapprich S, Daniels AD, Farkas O, Foresman JB, Ortiz JV, Cioslowski J, Fox DJ. GAUSSIAN 09 (Revision B.01). Wallingford, CT: Gaussian, Inc; 2010.
2. Kim K, Jordan KD. Comparison of density functional and MP2 calculations on the water monomer and dimer. J Phys Chem. 1994; 98: 10089–10094.
3. Stephens PJ, Devlin FJ, Chabalowski CF, Frisch MJ. Ab Initio calculation of vibrational absorption and circular dichroism spectra using densityfunctional force fields. J Phys Chem. 1994;98: 11623–11627.
4. S. Debnath, G. Mohiuddin, S. Turlapati, N. Begum, D. D. Sarkar, and N. V. S. Rao, Dyes and Pigments. 99, 447 (2013).

5. N. Begum, S. Turlapati, S. Debnath, G. Mohiuddin, D. D. Sarkar, and N. V. S. Rao, Liq. Cryst. 40, 1105 (2013).
6. R. Nandi, H. K. Singh, S. K. Singh, D. S. S. Rao, S. K. Prasad, B. Singh, R. K. Singh. Liq. Cryst. 2016, http://dx.doi.org/10.1080/02678292.2016.1269959.
7. 2. S. Kaur, V. Punjani, G .Mohiuddin, S. K. Pal. Orthogonal smectic and nematic ordering in three-ring polar bent-core molecules with anti-parallel arrangement. New J. Chem., 2017, 41, 5403-5411.
